# ABA Receptor Subfamily III Enhances Abscisic Acid Sensitivity and Improves the Drought Tolerance of *Arabidopsis*

**DOI:** 10.3390/ijms19071938

**Published:** 2018-07-02

**Authors:** Xiaoyi Li, Gaoming Li, Ying Li, Xiangge Kong, Liang Zhang, Jianmei Wang, Xufeng Li, Yi Yang

**Affiliations:** Key Laboratory of Bio-Resources and Eco-Environment of Ministry of Education, College of Life Sciences, Sichuan University, Chengdu 610065, China; yiendeavor@gmail.com (X.L.); 15196698789@163.com (G.L.); 2016222040068@stu.scu.edu.cn (Y.L.); 2016222040072@stu.scu.edu.cn (X.K.); liang.zhang113@gmail.com (L.Z.); wangjianmei@scu.edu.cn (J.W.); lixufeng0507@gmail.com (X.L.)

**Keywords:** abscisic acid, ABA signaling, abiotic stress, drought tolerance, ABA receptor

## Abstract

The phytohormone abscisic acid (ABA) regulates plant growth, the developmental process, and abiotic stresses. ABA signaling is induced in response to mediate plant acclimation to environmental challenges, including high salinity and drought. The ABA-binding receptors (RCAR/PYR1/PYL), composing of 14 members, are the core components of the ABA-signaling pathway. Here, we observed that the three subfamilies within the RCARs showed different expression patterns at the basal and exogenous ABA levels. Subsequently, we generated transgenic plants overexpressing subfamily III, *RCAR11–RCAR14*, respectively. The transgenic plants showed increased ABA sensitivity in seed germination and post-germination seedling establishment and root length. Further studies revealed that the overexpressing subfamily III transgenic plants enhanced drought resistance, increased water-use efficiency, and accelerated stress-responsive gene expression compared with the wild-type plants. These findings confirm that the subfamily III plays a key role in ABA-mediated developmental processes and, more importantly, is involved in drought tolerance in the ABA-dependent pathway.

## 1. Introduction

Abscisic acid (ABA) is an important plant hormone that regulates growth and development, and mediates abiotic stress, especially relating to drought and salinity [1,2,3]. The core ABA signaling could be considered as the RCARs-ABA-PP2Cs regulatory mechanism and the protein kinase SnRKs. In detail, under the absence or low levels of ABA, clade A PP2Cs interact with and dephosphorylate SnRK2s (SnRK2.2\2.3\2.6), reducing their activity. In contrast, ABA-binding receptors mediate the inhibition of PP2C activity, which releases the SnRK2s protein kinases and ultimately results in the activation of the ABA signaling pathway [4,5,6]. Subsequently, the SnRK2s directly phosphorylate the transcription factors that bind to the ABA-responsive promoter elements and the components of the machinery regulating stomatal aperture, which influences the drought tolerance [7,8].

In *Arabidopsis*, the regulatory component of ABA receptor (RCARs) family comprises 14 members that are divided into three subfamilies according to their sequence homology. RCARs show overlapping gene expression patterns, such as the expression of RCAR3/10/11/12/14 in the root vascular tissue [7,9] and all of them can activate ABA-responsive gene expressions using protoplast transfection assays [10]. However, there are different potentials to induce the ABA response. For example, the expression of RCAR4-7 and RCAR13 are too low to detect in the wild-type, while others are also different from the expression pattern [11,12]. The previous study found that RCAR11/12/14, members of the subfamily III, had little effect on the ABA signaling in the wild-type, whereas RCAR13 moderately activated the ABA response [9]. Previous studies reveal that at least two subfamilies of RCARs receptors are characterized by a different oligomeric state—that is, the dimeric state (RCAR11/12/13/14) and the monomeric state (RCAR3/8/9)—and the others are the yet unidentified homodimer or heterodimer in *Arabidopsis* [13]. Based on biochemical and structural data, the dimeric receptors show a higher dissociation constant for ABA than monomers; however, in the presence of exogenous ABA, both form the ABA-RCAR-PP2C ternary complexes [14].

Analysis of gene expression patterns and genetic analysis of different *rcar* (*pyr/pyl)* mutants have demonstrated that the function of RCARs proteins is not completely redundant [7,14,15]. RCAR1 and RCAR3 were more sensitive than other receptors in the regulation of protein phosphatase activity [15]. The single *rcar3 (pyl8)* mutant showed reduced sensitivity to the ABA-mediated root length inhibition [8], and the interaction of PLY8 and MYB77 promoted the lateral root growth independently of the core ABA-RCAR-PP2C signaling pathway to augment auxin signaling [16]. On the other hand, RCARs interact with ubiquitin ligases to attenuate ABA signaling, such as DDA1 allowing the ubiquitination of PYL8 [17], RSL1 targeting PYL4 [18], F-box E3 ligase RIFP1 mediating the RCAR3 stability [19], and AtRAE1 being involved in the degradation of RCAR1 [20]. In *Arabidopsis*, RCARs function show diversely and redundantly in the ABA signaling and drought-stress pathway [7,9], while the function is unknown until now. Therefore, we tend to clarity the roles of ABA receptors in *Arabidopsis*, including ABA signaling, drought tolerance and salt stress. 

To further understand the ABA receptors in ABA signaling, we analyzed the functionality of four members of RCARs in subfamily III of *Arabidopsis* in this study. The expressions of RCAR11/12/13/14 were not only detectable in the wild-type plant, but also significantly increased in the exogenous ABA. By comparison with wild-type (Col-0), the over expressions of RCAR11/12/13/14 showed more sensitivity to ABA-mediated inhibition of germination, root growth, drought tolerance, lower water loss, and induced ABA-mediated stomatal closure. The inhibitions of PP2C activity would increase only in the presence of ABA. This genetic evidence together with the ABA-dependent responsive genes analysis served to pinpoint the functions of the ABA receptors in subfamily III.

## 2. Results

**Figure 1:**
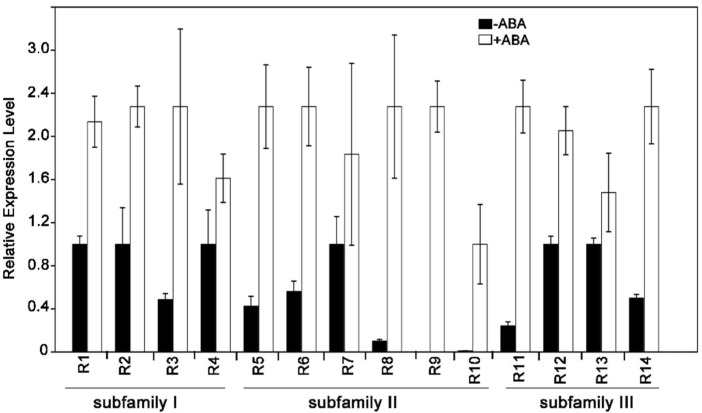
The quantitative RT-PCR of RCARs (R1-R14) genes. One-week-old plants were treated with 50 μM ABA and the total RNA was isolated at 0 or 3 h. *ACTIN2/8* was used as an internal control. Each value is the mean ± SE of the three independent biological experiments.

The response to ABA signaling was found to be quite variable and is stimulated by ectopic expression of RCARs in the presence of ABA [4,5,15,21]. To elucidate the core ABA signaling, we tested the transcript abundances of ABA receptors in the wild-type plants (WT) supplemented with or without ABA treatment. In the presence of exogenous ABA (50 μM), the expression levels of all 14 ABA receptors were induced in comparison with the plants without the treatment of ABA (Figure 1). Except for that, we also detected the RCARs expressions in the wild-type plant under drought and salt stress, the results indicating that RCARs also could be induced by these two stresses (Supplementary Figure S1). The findings indicated that the expressions of ABA receptors were induced by the exogenous ABA. However, there is no report about the physiological processes on the roles of subfamily III ABA receptors regulate ABA signaling and abiotic stresses.

**Figure 2:**
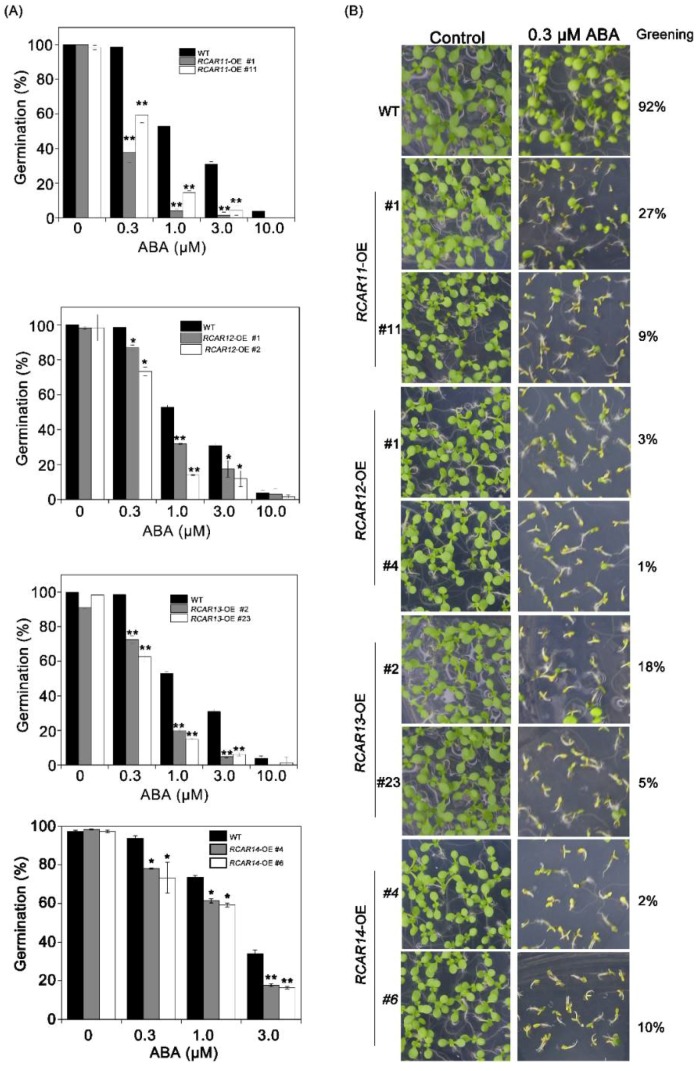
The germination and early seedling growth of the transgenic plants in response to ABA. (**A**) The seed germination rates of wild-type (WT) and *RCAR11/12/13/14* overexpression transgenic plants in the presence of ABA on the fourth day after stratification at 4 °C for 3 days; (**B**) Phenotypic analysis of WT and transgenic lines were grown on the MS medium with or without ABA and the green cotyledons were recorded 7 days after the stratification at 4 °C for 3 days. The green cotyledon percentage is indicated on the right. The experiments were repeated three times with similar results. Values are means ± SD (*n* > 100) of three independent experiments (* *p* < 0.05, ** *p* < 0.01, Student’s *t*-test).

**Figure 3:**
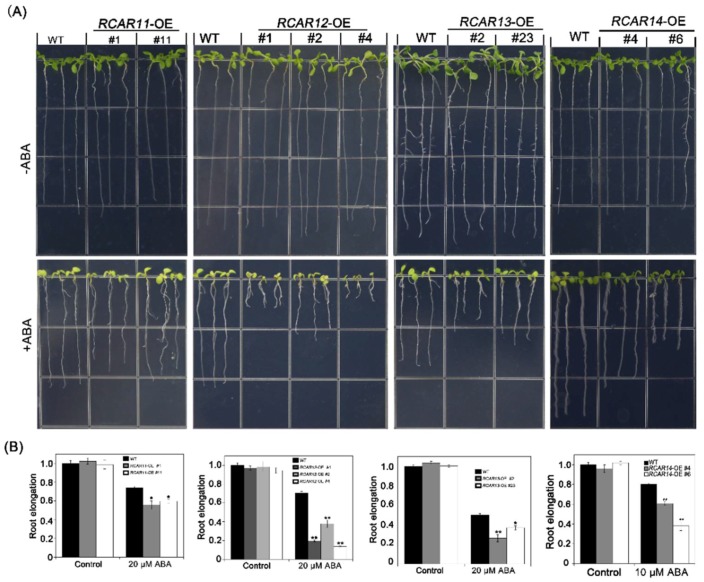
The overexpressions of the *RCAR11/12/13/14* transgenic plants are ABA-hypersensitivity in root architecture. (**A**) The inhibition of root growth of the RCAR11/12/13/14 transgenic lines in the presence of 10 μM ABA. The 3-day-old seedlings grown on the MS medium were transferred to 1/2MS plates lacking or supplemented with 10 or 20 μM ABA. The photographs were taken 7 days after the transfer; (**B**) The statistical analysis of the root length of the different genotypes described in (A). Values are means ± SD (*n* = 20). The experiments were repeated three times with similar results (* *p* < 0.05, ** *p* < 0.01, Student’s *t*-test).

The roles of subfamily III members were well known as ABA receptors, but there is no analysis of the detail function in ABA signaling. Therefore, we generated transgenic plants overexpressing *RCAR11*, *RCAR12*, *RCAR13,* and *RCAR14* in *Arabidopsis*, respectively (Supplementary Figure S1) and then carried on the phenotypic analysis. Our observation indicated that there was no significant difference between the WT and the transgenic plants in the germination and cotyledon greening rate in the absence of exogenous ABA (Figure 2). After 4 days, the seed germinations of *RCAR* transgenic plants were significantly lower than that of WT on MS medium supplemented the indicated ABA concentration. About 50% of WT seeds germinated, while only about 10–30% of *RCAR11/12/13/14*-OE seeds germinated (Figure 2). Similar results were observed in the analysis of cotyledon greening. The WT presented a higher ratio of the cotyledon greening than those of the overexpressing *RCAR* subfamily III transgenic plants. The cotyledon greening rate of WT was above 90%, while the cotyledon greening rates of *RCAR11/12/13/14* transgenic plants were lower than 30% (Figure 2). To further assess the regulation of the subfamily III ABA receptors in the root, a root growth assay was conducted. The results demonstrated that all *RCAR* transgenic plants showed significant inhibitions of root growth compared with WT in the 10 or 20 μM ABA-treated condition (Figure 3). Hence, the overexpression of ABA receptor subfamily III in *Arabidopsis* leads to plants being hypersensitive to ABA in the seed germination, post-germination growth, and root growth.

**Figure 4:**
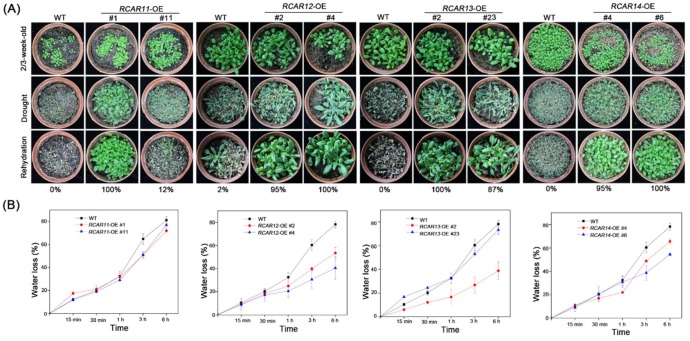
The RCAR11/12/13/14 are involved in the tolerance of *Arabidopsis* to drought stress. (**A**) Drought tolerance assay. Two-week-old seedlings were performed by withholding water for 13 days and subsequently re-watering for 2 days. The survival rates of WT and transgenic plants after drought and rehydration for 2 days. Four independent measurements were performed. The photographs show the representative measurement; (**B**) Water loss rates. Rosette leaves from 3-week-old plants were detached and the fresh weights were measured at the indicated time points. Three independent measurements were taken. Values are means ± SD (*n* = 6).

ABA signaling plays a key role in the plant response to drought [22,23]. Arabidopsis plants overexpressing *RCAR11*, *RCAR12*, *RCAR13*, and *RCAR14* may be tolerant to drought stress. To determine whether the sustained transcriptional upregulation of these ABA receptors affect drought tolerance, we exposed the wild-type and the transgenic plants to dehydration by withholding water for 13 d. There was no significant difference among them when growing 2 or 3-week old seedlings before drought tolerance. The results showed that WT nearly failed to survive, while the transgenic plants presented strong tolerance to drought stress with an above 80% survival rate, except for the *RCAR11*-OE #11 (12%). A water loss assay further confirmed our hypothesis. After 3 h, the loss of water of the detached leaves of WT (about 64%) was more than those of the transgenic plants, about 48% for *RCAR11-*OE, less than 30% for *RCAR12*-OE, about 20% for *RCAR13*-OE #2, and 40% for *RCAR14*-OE, respectively (Figure 4). These results indicate that the overexpressing ABA receptors of subfamily III in Arabidopsis led to the increased resistance of plants to drought stress. Except for the drought tolerance assay, the experiments were also carried out for the salt stresses, respectively. The result is shown in Supplementary Figure S3, and for the cotyledon greening rates of *R11*-OE, about 7% was significantly lower than that of other plants in the NaCl treatment. Moreover, the *R13*-OE transgenic plant (21.6%) was also insensitive to salt stress. In contrast, the *R12*-OE transgenic plant (100%) showed a slightly higher level of cotyledon greening than that of WT (91.67%). The greening rates of *R1*-OE, *R3*-OE were 75.87% and 86.11%, respectively. Taken together, the members of subfamily III play a positive role in drought tolerance, but not every receptor had the same function in abiotic stresses.

**Figure 5:**
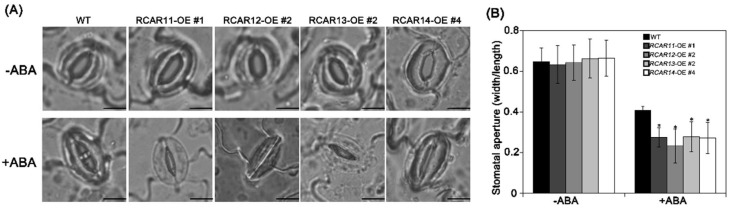
The RCAR11/12/1/14 induces ABA-mediated stomatal movement in transgenic *Arabidopsis*. (**A**) Stomatal closure of the WT and transgenic plants after 30 μM ABA treatment for 3 h; (**B**) Statistical analysis of the plants’ stomatal aperture (the ratio of width to length) shown in (**A**). Values are means ± SD (*n* = 50). * *p* < 0.05, Student’s *t*-test. Bar = 10 μm.

ABA is known to play an important part in inducing stomatal closure that mostly determines the rate of water loss under water-deficit conditions [7]. To elucidate the potential function of subfamily III members of ABA receptors in stomatal regulation, we measured the stomatal apertures of rosette leaves of Arabidopsis in response to ABA. After the treatment with 30 μM ABA for 3 h, the stomatal apertures (width/length) were reduced by about 24% (from 0.65 to 0.41) in WT and by about 35% in the overexpression of the subfamily III receptors plants compared to the treatment without ABA (Figure 5). This result clearly demonstrates that RCAR11/12/13/14 stimulates ABA-induced stomatal closure, which is consistent with the results of the drought stress and water loss.

**Figure 6:**
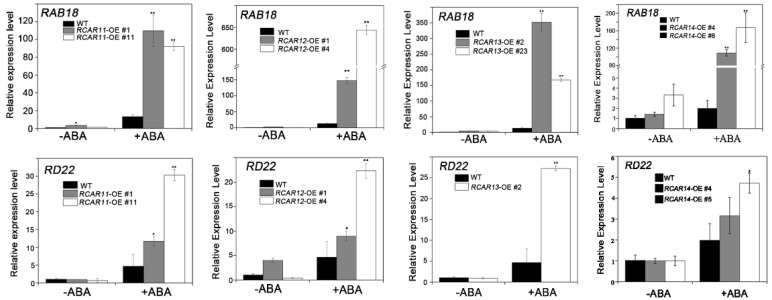
The RCAR11/12/1/14 attenuates the expression of several ABA-responsive genes. The relative expression levels of the ABA-responsive genes *RAB18* and the drought-responsive genes in the wild-type and *RCAR11/12/13* overexpression transgenic plants. The relative expression levels of *RAB18* and *RD22* in the WT and *RCAR11/12/13/14*-OE transgenic plant lines. Two-week-old seedlings were incubated in MS liquid medium with or without 50 μM ABA for 3 h. The transcriptional levels were determined by qRT-PCR analysis. Values are means ± SD (*n* = 3). *ACTIN2/8* was used as an internal control. The experiments were repeated three times with similar results (* *p* < 0.05, ** *p* < 0.01, Student’s *t*-test).

The previous studies showed that the over-expression of the ABA receptor would affect the transcript levels of genes downstream of the ABA signaling [24]. Therefore, the quantitative RT-PCR was employed to measure the transcript levels of the ABA-responsive genes-*RAB18* and the drought-responsive gene, *RD22*. In the absence of exogenous ABA, the expressions of *RAB18* and *RD22* were comparable between WT and the transgenic plants. However, the expressions of *RAB18* and *RD22* in *RCAR11-14* transgenic plants had significantly increased compared to that of WT after exogenous ABA treatment (Figure 6). These results suggest that the overexpression of *RCAR11*, *RCAR12*, *RCAR13*, and *RCAR14* affected the expression of those ABA signal-related genes, which modulate ABA response and drought tolerance. To test the effect of ABA receptors (RCAR11-RCAR14) on ABI1 activity, an enzymatic assay in vitro was employed. In the presence of ABA, the phosphatase activities of ABI1 was reduced to about 20% compared to that of ABI1 without the receptor, while in the absence of ABA, the phosphatase activities of ABI1 were no different, except for RCAR3 (Supplementary Figure S4).

## 3. Discussion

Upon binding to ABA, RCARs can induce conformational changes and then associate with PP2Cs, subsequently mediating the ABA signaling [25]. It is well known that receptors are characterized by the differences in oligomeric state, some being dimeric (RCAR11, RCAR12, RCAR13 and RCAR14), whereas others are monomeric (for example, RCAR3) [11,13,25]. In the present experiment, we confirmed that the expressions of the subfamily III receptors were moderately increased, the subfamily I receptors were efficiently active, and that subfamily II members were overall weakly active in ABA treatment. The result is consistent with that of the dimeric receptors being less sensitive to ABA than the monomeric receptors [13]. These results showed that the subfamily III receptors, in the absence of ABA, exist as inactive homodimers in the cells, unable to bind to PP2Cs (Supplementary Figure S4). However, in response to ABA binding, the homodimer interface of RCARs would change and then facilitate the subsequent dissociation of dimer [26].

There are 14 PYR/PYLs/RCARs members in *Arabidopsis*. The single-gene knockout mutants were reported to have incomparable phenotype with WT, indicating that the combination of several *plr*/*ply* loci was required to impair ABA signaling [7,8]. Therefore, the triple mutants (114) or more genes loss-of-function mutants (1124, 112,458) were needed to obtain a robust ABA-insensitive phenotype, which suggested a certain functional redundancy among *RCARs* [7]. Thus, we analyzed that phenotypes of the overexpressing RCARs subfamily III transgenic plants revealed their role as a positive regulator of ABA signaling.

Understanding ABA signaling is critical for the development of targeted approaches to improve crop performance under limited water supply. In this study, we demonstrated that the roles of subfamily III in the modulation of ABA signaling using overexpression transgenic plants. The overexpression of *RCAR11/12/13/14* promoted the low cotyledon greening rate and the germination in the presence of ABA, indicating that *RCAR11/12/13/14*-OE transgenic plants are more sensitive to exogenous ABA than WT (Figure 2). The root system of plants is one of the most sensitive organs in sensing the availability of water and nutrients or other adverse soil conditions [27,28]. The analysis of root growth after ABA treatment showed that the overexpression of transgenic plants was hypersensitive to ABA (Figure 3). The root length of *RCAR11*-OE transgenic plants was longer than that of other transgenic plants. One reason is that the transcription of *RCAR11* in *RCAR11*-OE transgenic plants only had about a seven-fold change, while that of other members had over twenty-fold changes in the correspondent transgenic plants. However, the seeds of *RCAR11*-OE were highly sensitive to ABA, that is, they were consistent with the high level of expression than that of the ABA receptors [7]. The interaction of ABA with other hormones, such as JA [29], that regulates root length will be another possible explanation. These results suggested that *RCAR11/12/13/14* exhibited ABA hypersensitivity in all the major ABA response in *Arabidopsis*, including the ABA-induced inhibition of seed germination, cotyledon greening, and root length.

ABA receptors could promote drought resistance, such as PYL9 (RCAR1) [30]. In comparison with wild-type plants, the overexpression of the *RCAR11/12/13/14*-OE transgenic plants had relatively high survival rates (Figure 4A). Consistently, with the drought tolerance, the *RCAR11/12/13/14*-OE transgenic lines displayed significantly reduced water loss rates (Figure 4B). Except for the drought tolerance, the ABA receptors also involved salt-responsive signaling (Supplementary Figure S3). Furthermore, the overexpression of *RCAR11/12/13/14* induces stomatal closure, respectively. We further confirmed that overexpression of *RCAR11/12/13/14* could increase the expression levels of the ABA-responsive gene (*RAB18*) and the drought-responsive gene (*RD22*). The inhibition of the PP2C phosphatase activity was consistent with that of the family III ABA receptors requiring ABA and then PP2C, while the monomeric state receptors are ABA-independent [10,26]. Taken together, our findings confirmed the family III ABA receptors as positive regulators of the ABA-mediated drought stress responsiveness in *Arabidopsis*.

In summary, we have shown that the overexpression of the family III receptors results in the hypersensitivity of plants to ABA and enhances the drought tolerance of plants. The previous analysis showed that the overexpression of *RCAR3*, *RCAR6*, and *RCAR10* also increased the water-use efficiency and resulted in increase of seed productivity [2,16,24]. We confirmed that the ABA receptors are the core component of the ABA signaling pathway in *Arabidopsis*, especially when the plants suffer from abiotic stresses.

## 4. Materials and Methods

### 4.1. Plant Materials and Growth Conditions

Arabidopsis ecotype Col-0 was used as the wild-type control in this study. To generate transgenic plants overexpressing *RCAR11*, *RCAR12*, *RCAR13*, and *RCAR14*, the open reading frames (ORF) of *RCARs* were amplified by PCR using specific primers (Supplementary Table S1) and inserted into the pBI121 vector at the *Bam*HI and *Sac*I sites under the driving of the cauliflower mosaic virus 35S promoter. After the constructs were introduced onto the GV3101 strain of *Agrobacterium tumefaciens*, the constructions were transformed by the floral infiltration into Col-0 plants. The transgenic plants were identified by the Kanamycin resistance and their mRNA levels were verified with RT-PCR assays. The T3 homozygous seeds of the transgenic plants were used for further analysis.

*Arabidopsis* plants were routinely grown under greenhouse conditions in pots containing a mixture of vermiculite and soil (1:3). For the plate culture, the seeds were surface sterilized by treatment with 75% ethanol for 5 min, followed by commercial bleach (20%) for 15 min, and finally, four washes with sterile distilled water. The stratification of the seeds was conducted in the dark at 4 °C for 3 days. Then seeds were sown on an MS medium containing 2% sucrose and 0.8% agar, pH 5.7 with KOH. All the plants were incubated in the growth chamber at 22 °C under a 16-h-light/8-h-dark photoperiod at 80 to 100 μE m^−2^ s^−1^.

### 4.2. RNA Extraction and Quantitative Real-Time PCR (RT-PCR) Analysis

The total RNA was extracted from 10-d-old *Arabidopsis* seedlings treated with 50 μM ABA for 3 h using TRIzol RNA reagent (TaKaRa, Bejing, China). The cDNA was synthesized using the PrimeScript RT reagent Kit with a gDNA Eraser (TaKaRa, Beijing, China), following the manufacturer’s instruction. PR-PCR was carried out on a CFX96 Touch™ Real-Time PCR detection system (Bio-Rad, Hercules, CA, USA) with iQ™ SYBR Green Supermix and gene-specific primers (Supplementary Table S1). All reactions were performed in triplicates with the following cycling conditions: 95 °C for 5 min; 45 cycles each at 95 °C for 20 s and 52 °C for 20 s, and 72 °C for 20 s. Actin2/8 was used as an internal standard.

### 4.3. Phenotype Analysis

The cotyledon greening and seed germination assays were carried out as described in References [19,31,32]. About 60 seeds from the wild-type plants and the transgenic plants were disinfected and sown on an MS medium supplement with the indicated ABA or NaCl concentration after stratification. The germination rate was determined after 4 days. The cotyledon greening rate was recorded at the percentage of seeds that developed green expanded cotyledons and the first pair of true leaves at 7th Day.

For the root growth assay, the seeds from each line were germinated vertically on the MS medium for 3 days after the stratification in the dark at 4 °C for 3 days. Then, 20 seedlings of each line sharing similar root lengths were transferred to an MS medium supplemented with or without 10 or 20 μM ABA in the vertical position. The root length was determined after the transfer for 7 days.

For the drought tolerance assay, 2-week-old plants were subjected to progressive drought by withholding water for 13 days, re-watered for 2 days, and then determined the survival rate. For the water loss assay, the rosette leaves of 3-week-old plants were detached, and then placed on a Petri’s dish and the weight at the indicated time was measured (0 min, 15 min, 30 min, 1 h, 3 h, and 6 h).

For the determination of the water loss rate, the water loss was measured by the methods described by References [19,30]. Six rosette leaves per plant were excised from 3-week-old plants. The leaves were then exposed to ambient conditions and the loss of weight was recorded at the indicated time.

For the stomatal aperture measurements, 4-week-old seedlings were put in the darkness for 24 h the stomata to close. Then, epidermal strips of the rosette leaves were peeled and immersed in the opening buffer (10 mM MES-KOH (pH 6.15), 10 mM KCl, and 50 μM CaCl_2_ with or without 30 μM ABA) for 3 h exposed to continue light [20,33]. The stomatal apertures were examined and photographed with a fluorescence microscope (DMI6000B, Leica, Wetzlar, Germany). More than 50 stomatal apertures (Length/width) from each line were measured with ImageJ quantification.

### 4.4. Statistical Analysis

The data are represented as means ± SD. The statistical analysis was performed using Student’s *t*-test. The values of *p* < 0.05 were considered significant, and the values of *p* < 0.01 were considered more significant.

## Supplementary Materials

The following are available online at http://www.mdpi.com/1422-0067/19/7/1938/s1.
