# Supplementary material for: ABA Receptor Subfamily III Enhances Abscisic Acid Sensitivity and Improves the Drought Tolerance of Arabidopsis
Source: Int J Mol Sci. 2018 Jul 2;19(7):1938. doi: 10.3390/ijms19071938 (PMC6073838; doi:10.3390/ijms19071938)
Supplement: Supplementary file 1 [file ijms-19-01938-s001.pdf]

Supplementary File

ABA Receptors Subfamily III Enhances Abscisic Acid Sensitivity and Improves Drought Tolerance of Arabidopsis

Methods

In vitro PP2C Phosphatase Assay

RCAR3 and RCAR11-RCAR14 were cloned into the pET28a expression vector with 6 × His tag fused at the C-terminal side and the primers were used as shown in table S1. ABI1 (residues 117-434) was cloned into pGEX-6p-1 expression vector and transformed into *E. coli* strain *Rosetta* (DE3). All proteins were purified as described previously (Zhang *et. al.*, 2018).

Reactions were performed in a 5× reaction buffer containing 250 mM imidazole, pH 7.2, 1 mM EGTA, 25 mM $MgCl_2$, 0.1% β-mercaptoethanol and 0.5 mg/ml BSA. 0.3 μM ABI1 (residues 117-434), 3 μM RCARs proteins and 10 μM (+)-ABA (Sangon Biotech, Shanghai, China) were added if required. After incubation with peptide substrate (RRA(pT)VA) at 30 °C for 20 min, the reaction was stopped by addition of 50 μL molybdate dye. Absorbance at 630 nm was measured after 20 min at room temperature. The values shown were normalized to the control (ABI1) as 100% activity.

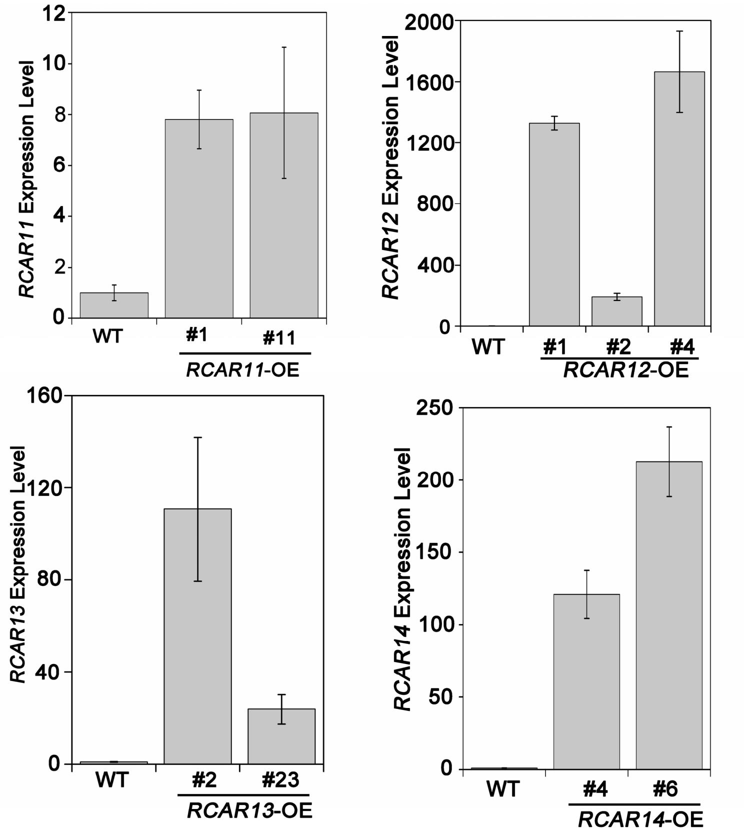

Figure S1. Quantitative RT-PCR analysis of *RCAR11-RCAR14* gene expression in overexpression transgenic lines. Expression level in wild-type Col-0 (WT) and all the genetic materials was normalized to that of *ACTIN2/8* and the expression levels of *RCAR11-RCAR14* in WT were set to 1. Each value is the mean ± SE of the three independent biological experiments.

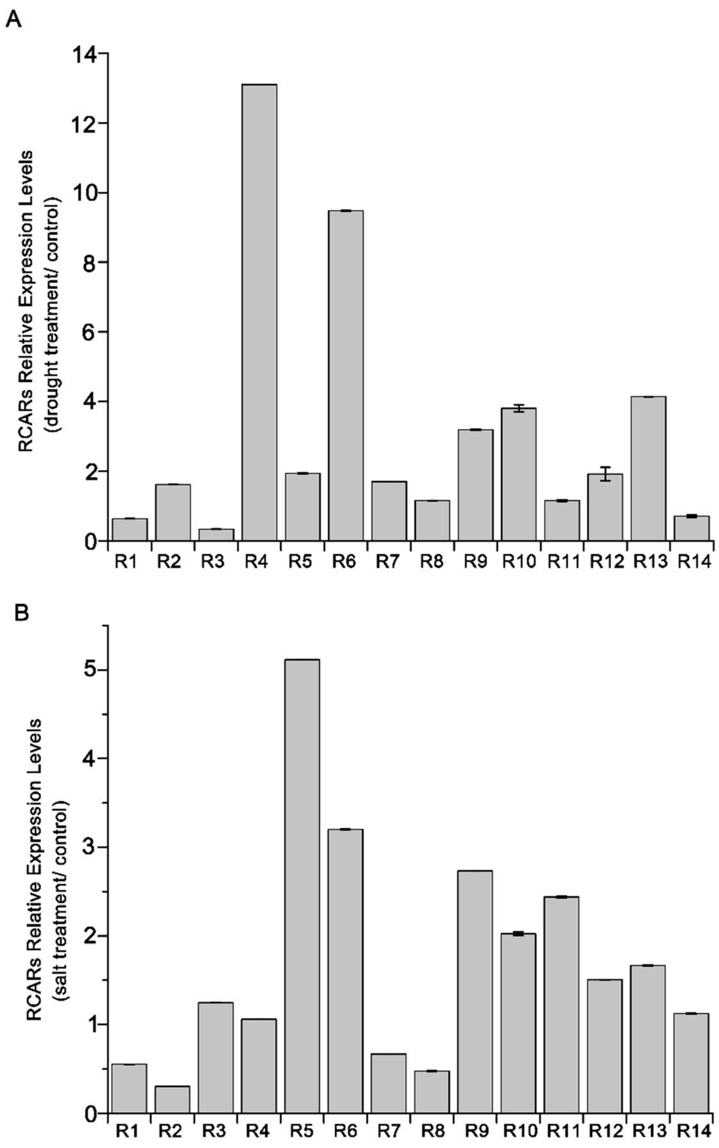

Figure S2. The quantitative RT-PCR of RCARs (R1-R14) genes. **A**. Two-week-old the wild type plants were detached and the total RNA was isolated at 0 and 1 h. **B**. One-week-old the wild type plants were treated with 150 mM NaCl and the total RNA was isolated at 0 and 6 h, respectively. *ACTIN2/8* was used as an internal control. Each value is the mean ± SE of the three independent biological experiments.

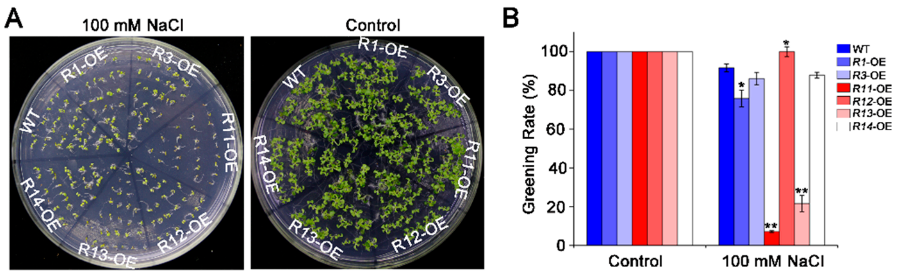

Figure S3. Effect of RCARs responding to salt during early seedling development. **A**. Growth status of the transgenic seedlings and wild type grown on MS medium with and without 100 mM NaCl for 5 days and 7 days, respectively. **B**. Statistical analysis of cotyledon greening rate of seedlings grown on MS medium with and without 100 mM NaCl for 5 days and 7 days, respectively.

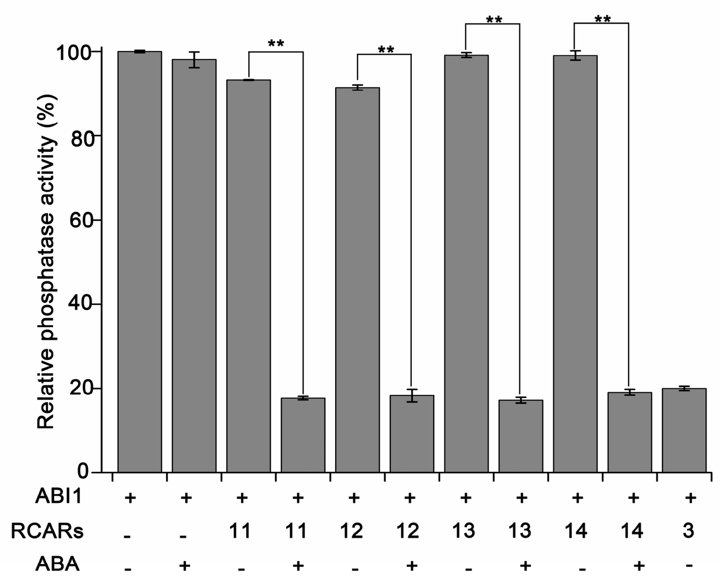

Figure S4. Effects of various phosphorylation forms of RCARs on the inhibition of ABI1 activity in the absence or presence of ABA. The concentrations of each protein component were 0.3 μM for ABI1, 3 μM for WT and phosphor-mutants of RCARs, and 10 μM (+)-ABA. Error bars represent SD (n=3). **P < 0.01, Student's t-test.

Table S1. Primers for transgenic plant construction and RT-PCR analysis.

	Genes	Primer sequences (5'-3')
Primers for transgenic plant construction	RCAR11	F (*BamH*I): CGGGATCCATGCCTTCGGAGTTAACACCA
		R (*Sac*I): CGAGCTCCGTCACCTGAGAACCACTT
	RCAR12	F (*BamH*I): CGCGGATCCATGGCGAATTCAGAGTCCTC
		R (*Sac*I): CGAGCTCCCTAACCTGAGAAGAGTTGT
	RCAR13	F (*BamH*I): CGGGATCCATGAATCTTGCTCCAATCCA
		R (*Sac*I): CGAGCTCGGTCGGAGAAGCC
	RCAR14	F (*BamHI*): CGGGATCCATGAGCTCATCCCCGG
		R (*SacI*): CGAGCTCTTCATCATCATGCATAGGTG
Primers for PP2C phosphatase assay	RCAR12	F (*Sac*I): CGAGCTCATGGCGAATTCAGAGTCCTC
		R (XhoI): CCGCTCGAGCCTAACCTGAGAAGAGTTGT
	RCAR13	F (SacI): CGAGCTCATGAATCTTGCTCCAATCCA
		R (XhoI): CCGCTCGAGGGTCGGAGAAGCC
	RCAR14	F (*BamHI*): CGGGATCCATGAGCTCATCCCCGG
		R (*Xho*I): CCGCTCGAGTTCATCATCATGCATAGGTG
Primers for RT-PCR	RCAR1	AAGGCGGCACGGCGAT
		ACGGTTTGTATTTCTGCGG
	RCAR2	CACTGGTGCGGAGATTTG
		GATGTTGATACCGAGGATGT
	RCAR3	GGGATTGAGAACTTGACGA
		AAACGGCTTATACTTCTGTG
	RCAR4	CGGAGACGATAGACGGA
		GACGCTCGGTAACATCTG
	RCAR5	TTCCAGCGGAGTTCAGC
		CCGGCACATCCACCAC
	RCAR6	CGATCTTCCGGCGAGTTT
		AGTATTTCCTTCCGGCACAT
	RCAR7	TAGCGTAGTCGAGACCATT
		GGGAAGCCGGAGACTAA
	RCAR8	CGCGGTATGCATGTCCC
		ACGATACGGCACTGTCTG

RCAR9	TATCCCCACCACCATCAG
	AGCGGCTTAGGATCGAC
RCAR10	GCTCCGCCGTTATTCAAG
	GCTAGCGGCGGGGAG
RCAR11	ATCGTCATCAGTGGATTA
	TAATTCGTCAGCCTATGT
RCAR12	TGCTGATACGGTTATTAGATTGA
	GAGAAGAGTTGTTGTTGTTGTT
RCAR13	GTGTATAGTGTGGTATTGG
	GCGAGATTCTGTAGATTC
RCAR14	GTCGGTCAATGAGTTCTT
	TAGTGTCTTCCTCTGTGTT
RD22	AGGAGCAAACCCTTTCGTGT
	CGTTTCAACGTCTCCGAAAA
Actin2/8	AAGATCAAGGAGAAGTTGCCAGG
	GTAAACAACACACATCGCAGGACG
RAB18	AAGATCAAGGAGAAGTTGCCAGG
	GTAAACAACACACATCGCAGGACG

© 2018 by the authors. Submitted for possible open access publication under the terms and conditions of the Creative Commons Attribution (CC BY) license (http://creativecommons.org/licenses/by/4.0/).
